# Inhibition of T cell immunoglobulin and mucin-1 (TIM-1) protects against cerebral ischemia-reperfusion injury

**DOI:** 10.1186/s12964-019-0417-4

**Published:** 2019-08-22

**Authors:** Yueying Zheng, Liqiang Wang, Manli Chen, Lu Liu, Aijie Pei, Rong Zhang, Shuyuan Gan, Shengmei Zhu

**Affiliations:** Department of Anesthesiology, The 1st Affiliated Hospital, School of Medicine, Zhejiang University, 79# Qingchun Road, 310003 Hangzhou, Zhejiang Province People’s Republic of China

**Keywords:** The T cell Ig domain and mucin domain (TIM)-1, Cerebral ischemia-reperfusion injury, MCAO, T cell

## Abstract

**Background:**

The T cell Ig domain and mucin domain (TIM)-1 protein expressed on the surface of Th2 cells regulates the immune response by modulating cytokine production. The present study aimed to investigate the role and possible mechanism of TIM-1 in cerebral ischemia-reperfusion injury.

**Methods:**

Western blot was used to detect TIM-1 and apoptosis-related protein expression, whereas TIM-1 mRNA was examined using quantitative real-time reverse transcription PCR. Flow cytometry and a TdT-mediated biotin-16-dUTP nick-end labeling (TUNEL) assay were used to detect the percentage of apoptotic cells and a pathological examination was performed. The migration of neutrophils and macrophages was analyzed by immunohistochemistry.

**Results:**

Our results suggest that TIM-1 expression was transiently increased 24 h or 48 h following middle cerebral artery occlusion (MCAO)/reperfusion. The infarct size was markedly increased in MCAO, whereas treatment with a TIM-1-blocking mAb could reduce the infarct size. TIM-1 blocking mAb effectively reduced the number of neutrophils, macrophage functionality, cytokine (i.e., IL-6, IL-1β, and TNF-α) and chemokine (i.e., CXCL-1 and CXCL-2) production in the brain tissue. The effect of in vitro T cell damage on neurons was significantly reduced following treatment with a TIM-1 blocking mAb or the knockdown of TIM-1 in co-cultured T cells and neurons.

**Conclusion:**

Take together, these results indicated that TIM-1 blockade ameliorated cerebral ischemia-reperfusion injury. Thus, TIM-1 disruption may serve as a novel target for therapy following MCAO.

## Background

Stroke is the leading cause of severe long-term neurological disability in developed countries, and is associated with an incidence rate of approximately 0.25–4% and a mortality rate of about 30% [1]. Moreover, approximately 85% of strokes are initially ischemic, typically caused by atherothrombotic or cerebral arterial embolism [2]. Multiple factors are associated with high risk of stroke, including hypertension, atherosclerosis, type 2 diabetes, hyperlipidemia, hyperhomocysteinemia, smoking, and drinking [3]. Extensive evidence indicates that ischemia is accompanied by a series of neurological events (e.g., hypoxia, oxidative stress, and inflammatory response [4]) and eventually results in neuronal cell acute necrosis, apoptosis, and autophagy in the ischemic brain [5, 6]. To date, thrombolysis by intravenous recombinant tissue plasminogen activator (tPA) is the only clinically effective therapeutic strategy for ischemic stroke [7]. Therefore, additional effort is required to identify the delicate mechanism of cerebral stroke underlying stroke-induced cellular death and neurological dysfunction.

During the acute phase of ischemic reperfusion (I/R) injury, there are multiple waves of cellular infiltration, consisting of macrophages, neutrophils, and lymphocytes [8]. Brain-infiltrating T cells have also been widely reported in stroke and animal models of stroke and are thought to have acute detrimental and delayed protective effects [9]. The family of T cell immunoglobulin and mucin domain (TIM) proteins are type 1 transmembrane proteins, including eight mouse proteins (TIM-1 - TIM-8) and three members (TIM-1, TIM-3, and TIM-4) in humans are expressed on various immune cells and can also mediate coinhibitory and costimulatory signals [10, 11]. TIM-1 is an important susceptibility gene for asthma and allergy preferentially expressed on T-helper 2 (Th2) cells and functions as a potent costimulatory molecule for T cell activation [10]. Although T lymphocytes are key contributors to the acute phase of cerebral ischemia reperfusion injury, the relevant T cell-derived mediators of tissue injury remain unknown. It has been previously reported that TIM-1 ligation in combination with the T cell receptor provides a positive co-stimulatory signal, resulting in enhanced T cell proliferation, cytokine production, and the abrogation of tolerance [12, 13]. Using an antagonistic anti-TIM monoclonal antibody (mAb) [14], it was shown that a TIM-1 blockade prolongs allograft survival by downregulating Th1 cells and promoting Th2-mediated alloresponses [15]; however, no reports have studied the role of TIM-1 in stroke.

In the present study, we confirmed that the suppression of TIM-1 plays an important protective effect on cerebral ischemia-reperfusion injury. Our research suggests a vital role for TIM-1 in MCAO injury, indicating a potential target for the treatment for cerebral ischemia-reperfusion injury.

## Materials and methods

### Animals

Six-week-old C57BL/6 J mice (20 g - 25 g, male) were purchased from the Experimental Animal Center of Zhejiang University School of Medicine. All mice were housed in an environmentally controlled room under a 12 h light/dark cycle with ad libitum access to food and water. All animal experiments were approved by the First Affiliated Zhejiang Hospital, Zhejiang University of Medical Ethics Committee, the Medical Faculty Ethics Committee of the First Affiliated Zhejiang Hospital, Zhejiang University in accordance with the National Institutes of Health Guide for Care and Use of Laboratory Animals (NIH Publications, No. 8023, revised 1978).

### Middle cerebral artery occlusion (MCAO)/reperfusion model

Middle Cerebral Artery Occlusion (MCAO) was established using previously published methods, as described previously [16, 17]. A total of 24 C57BL/6 J mice were randomly divided into four groups (*n* = 6): Blank group, MCAO 24 h, MCAO 48 h, and TIM-1 mAb [18] + MCAO 48 h (an infusion containing a blocking monoclonal antibody against TIM-1 (0.5 mg/mouse i.v.; Bio X Cell, West Lebanon, NH, USA) at 1 h prior to the induction of ischemia). In brief, 4% chloral hydrate (Sigma, St. Louis, MO, USA) was used to anesthetize the mice and a 6–0 silicone-coated nylon monofilament (Doccol Corp., Redlands, CA, USA) was inserted into the left common carotid artery to occlude the MCA origin. After 1 h, the suture was removed. In the blank groups, filaments were prepared and inserted into the left common carotid artery. The mice were anaesthetized and decapitated to obtain the brain.

### Measurement of the cerebral infarction area

After 24 h of reperfusion, 1 mm-thick coronal sections of the brain were immersed in a 0.05% 2,3,5-triphenyltetrazolium chloride (TTC) solution for 30 min at 37 °C. The total area of each brain section and the infarcted region were quantified using the Image J software program (v1.46, National Institutes of Health). The infarct volume was corrected for edema as previously described [19, 20].

### Bederson score

Normal mice exhibited a head lift and the two front paws extended to the table top when the mouse tail was raised to 5 cm above the table top. The brain injury mice displayed buckling of the contralateral forelimbs to the brain injury. The postural changes from mild flexion, elbow extension, shoulder abduction, severe wrist and elbow flexion, and shoulder internal rotation abduction were observed. The Bederson score was divided into four grades according to the posture reflex and shoulder lateral thrust test results of the mice. 0 points: no neurological deficit symptoms were observed; 1 point: buckling of the contralateral forelimbs of the infarct hemisphere when the tail was suspended, but not with other abnormalities; 2 points: when the tail is suspended, the contralateral forelimb of the infarct hemisphere is flexed, and the opposite side of the infarct hemisphere is pushed by hand, and its resistance is reduced, but it does not rotate when freely moving; 3 points: the same two points behavior, and when you are free to move, turn to the side of the squat.

### Enzyme-linked immunosorbent assay

The level of lactate dehydrogenase (LDH), tumor necrosis factor alpha (TNF-α), macrophage inflammatory protein-1α (MIP-1α), NO, and interleukin (IL-6) was determined using a commercial ELISA kit according to the manufacturer’s instructions (R&D Systems, Minneapolis, MN, USA).

### Immunohistochemical analysis

Mouse liver tissues were fixed in a 10% formalin solution for 24 h, embedded in paraffin, sliced, dewaxed, and hydrated according to conventional techniques. The samples were incubated in 5% fetal bovine serum for 30 min at room temperature followed by an overnight incubation at 4 °C with the primary antibodies: CD3, Ly6G, and CD68 (Abcam, Cambridge, MA, USA). The samples were incubated for 30 min at 37 °C after the addition of a horseradish peroxidase-conjugated secondary antibody (anti-rabbit-HPR, 7074, Cell Signaling Technology, Danvers, MA, USA). The samples were then treated with diaminobenzidine for coloration. The cell nuclei were counterstained with hematoxylin, and the samples were dehydrated in a gradient series, vitrified with dimethylbenzene, and finally mounted with neutral balsam.

### TdT-mediated biotin-16-dUTP nick-end labeling (TUNEL) assay

A TUNEL assay was performed using the One-Step TUNEL Apoptosis Assay kit (Roche, Basel, Switzerland) to detect apoptotic cells in the mouse livers. Briefly, 4-μm-thick paraffin sections were deparaffinized, hydrated, treated with proteinase K for 20 min, and subsequently incubated with a mixture of a fluorescent labeling solution of dUTP and the TdT enzyme at 37 °C for 1 h in a humidified atmosphere. As a positive control, the sections were incubated with DNaseI for 10 min at room temperature (25 °C) prior to the fluorescent labeling procedure. Negative controls were incubated with dUTP for 10 min at room temperature (25 °C). The samples were subsequently treated with diaminobenzidine, counterstained with hematoxylin (to identify the cell nuclei), dehydrated in a gradient series, vitrified with dimethylbenzene, and finally mounted with neutral balsam.

### Real-time polymerase chain reaction

Total RNA was isolated using Trizol reagent (Invitrogen, Carlsbad, CA, USA), and the RNA concentration was measured using spectrophotometry. Single-stranded cDNA was synthesized using a cDNA synthesis kit (Takara, Kyoto, Japan) according to the manufacturer’s instructions. Reverse transcription-polymerase chain reaction assays were performed using Applied Biosystems SYBR Green Mix kits (Applied Biosystems, CA, USA). GAPDH was used as a housekeeping gene. The results were presented as the ratio of the target gene to GAPDH mRNA (sense and antisense). The following primers were used in the present study:

IL-6:

Forward 5′-CCACTTCACAAGTCGGAGGCTTA-3′

Reverse 5′- CCAGTTTGGTAGCATCCATCATTTC-3′;

CXCL-1:

Forward 5′- CTGGGATTCACCTCAAGAACATC-3′

Reverse 5′-CAGGGTCAAGGCAAGCCTC-3′

CXCL-2:

Forward 5′-CCAACCACCAGGCTACAGG-3′

Reverse 5′- GCGTCACACTCAAGCTCTG − 3′;

Tim-1:

Forward 5′-ACATATCGTGGAATCACAACGAC-3′

Reverse 5′- ACAAGCAGAAGATGGGCATTG -3′;

IL-1β:

Forward 5′-AAATCTCGCAGCAGCACAT-3′;

Reverse 5′-CACACACCAGCAGGTTATCA-3′;

TNFα:

Forward 5′-TATGGCCCAGACCCTCACA-3′

Reverse 5′- GGAGTAGACAAGGTACAACCCATC-3′.

### Western blot analysis

The total protein was extracted and quantified using the bicinchoninic acid method. Equal amount of each protein (40 μg/lane) was separated on 10% SDS-PAGE gels and transferred to PVDF membranes (Millipore, Billerica, MA, USA). The membranes were blocked with 5% non-fat milk in TBST buffer for 1 h and then incubated overnight with the following primary antibodies: TIM-1, Bax, Bcl-2, Bcl-xl (dilution 1:1000, Abcam, Cambridge, MA, USA), and GAPDH (Cell Signaling Technology, Danvers, MA, USA). After washing twice in TBST, the membranes were incubated with a horseradish peroxidase-conjugated secondary antibody (anti-rabbit-HPR, 7074, Cell Signaling Technology, Danvers, MA, USA) at a 1:2000 dilution. Specific bands were visualized using an enhanced chemiluminescence detection kit (GE Healthcare, Piscataway, NJ, USA).

### Flow cytometric analysis

Peripheral blood mononuclear cells were isolated by centrifugation on a Ficoll (Lymphoprep, PAA, Nycomed, Oslo, Norway) gradient from buffy coat preparations obtained from mice, and then washed twice with PBS. The cells were then incubated with a primary anti-TIM-1 antibody (Abcam, Cambridge, MA, USA) for 30 min at 4 °C, washed twice with PBS, and incubated with a secondary FITC-conjugated antibody (Abcam, Cambridge, MA, USA) for 30 min at 4 °C. The cells were subsequently washed twice with PBS, incubated with 600 μL PBS at room temperature, and examined using flow cytometry.

### Statistical analysis

All experimental values are expressed as the mean ± SD. Statistical analysis was performed using GraphPad Prism 5.0 software (GraphPad Software, San Diego, CA, USA). Statistical analysis was performed using a *t*-test if only two conditions were analyzed. A one-way ANOVA with a Bonferroni post-test was used for multiple comparisons. A *P* value of < 0.05 was considered to be statistically significant.

## Results

### TIM-1 expression was upregulated in MCAO

**Fig. 1:**
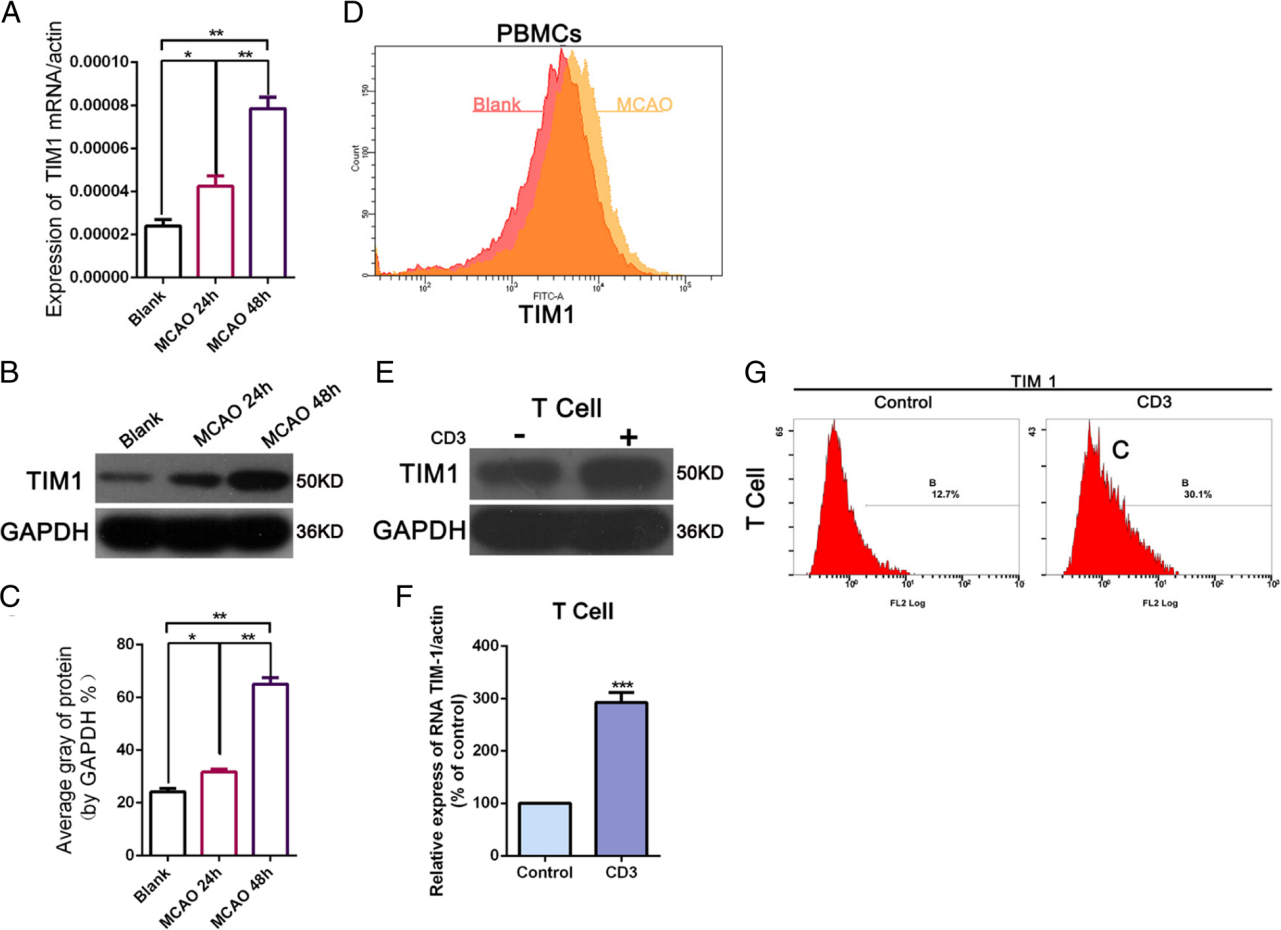
TIM-1 expression is upregulated in MCAO. **a** TIM-1 mRNA expression was detected by quantitative reverse-transcription PCR (qRT-PCR) at 24 h and 48 h after MCAO. *p < 0.05; **p < 0.01 vs Black. **b**-**c** Western blot detection of the level of TIM-1 protein expression 24 h and 48 h after MCAO. *p < 0.05; **p < 0.01; ***p < 0.001 vs Black. **d** Flow cytometry was used to measure the level of TIM-1 expression in the PBMCs. **e**-**f** TIM-1 protein expression and miRNA were examined by Western blot and qRT-PCR following treatment with or without the CD3 monoclonal antibody-stimulated T cells cultured in vitro. ***p < 0 .001 vs Control. **g** The level of TIM-1 expression was detected by flow cytometry following treatment with or without in vitro CD3 monoclonal antibody stimulation

To determine the effect of TIM-1 on the MCAO, we detected the level of TIM-1 mRNA and protein expression after MCAO at 24 h and 48 h by qRT-PCR and Western blot. The results found that the expression of TIM-1 mRNA and protein was significantly upregulated 24 h and 48 h after MCAO; moreover, the longer the MACO time (48 h), the higher the expression of TIM-1 (Fig. 1a-c). We then determined the changes of TIM-1 in PBMCs by flow cytometry analysis and found that TIM-1 expression was increased in the MACO group compared with the Control (Fig. 1d). Later, we used CD3 to activate the T cells in vitro, and a Western blot and qRT-PCR were used to examine the expression of TIM-1 following treatment with or without CD3. The results showed that the expression of TIM-1 mRNA and protein was upregulated after CD3 monoclonal stimulation (Fig. 1e-f). Flow cytometry showed that TIM-1 expression was increased after CD3 monoclonal stimulation (Fig. 1g). These results indicate that the high expression of TIM-1 was correlated with T cell activation following MACO.

### TIM-1 blocking mAbs effectively protect against brain tissue damage in an MCAO model

**Fig. 2:**
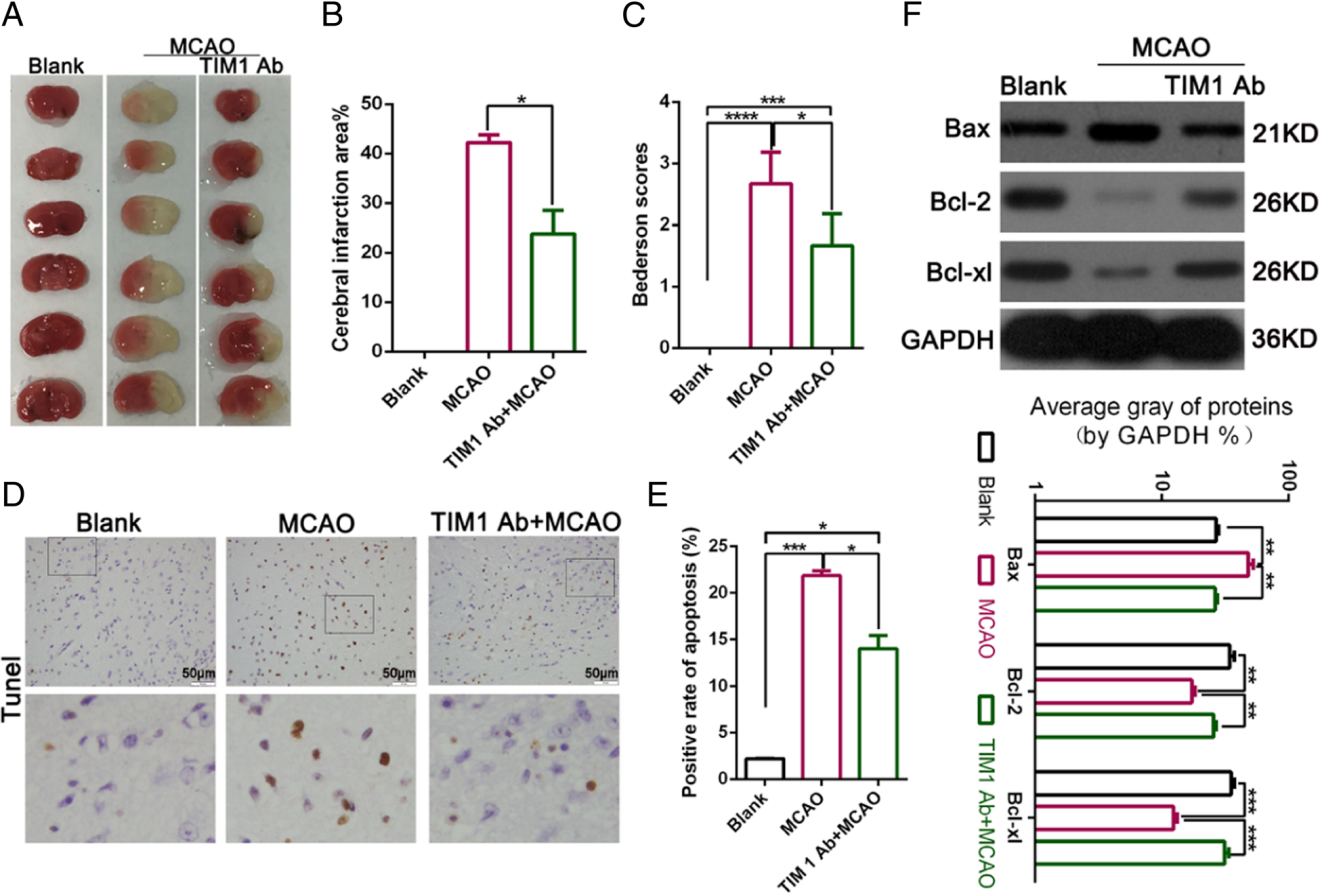
TIM-1 blocking antibody effectively protects against brain tissue damage in a model of MCAO. **a**-**b** The infarct size was measured in the Black, MCAO, and TIM-1 Ab + MCAO groups by TTC staining. **c** Bederson scores of the **mice** after MCAO following treatment with or without TIM-1 Ab. **d**-**e** Cellular apoptosis was examined using TdT-mediated biotin-16-dUTP nick-end labeling (TUNEL) assay (magnification: 400×; *P < 0.05; **P < 0.01; ***p < 0.001 vs Black. **f** Western blot detecting the level of apoptosis-related protein expression (Bax, Bcl-2, and Bcl-xl)

To further explore whether a TIM-1 blockade had a protective role in an MCAO model, we used TCC to examine the infarct area. The TTC staining results indicated that MCAO resulted in an increased infarct size; however, treatment with an anti-TIM-1 mAb induced a less severe infarct than the MCAO group and led to a significant decrease in the infarct area compared with MCAO (Fig. 2a-b). We then used the Bederson Score to score all the functional neurological deficits in MCAO following treatment with or without the anti-TIM-1 mAb. The results showed that after inhibiting TIM-1, the Bederson score was significantly decreased (Fig. 2c). Moreover, the TUNEL analysis revealed that inhibiting TIM-1 could significantly decrease the rate of apoptosis in MCAO (Fig. 2d-e). Almost immediately, inhibiting TIM-1 with an anti-TIM-1 mAb significantly down-regulated the level of Bax protein and up-regulated Bal-2 and Bcl-xl compared with MCAO (Fig. 2f). Our data indicates that the anti-TIM-1 mAb effectively protected against brain tissue damage in the MCAO model.

### Inhibiting TIM-1 effectively reduced neutrophil and macrophage function in the brain tissue

**Fig. 3:**
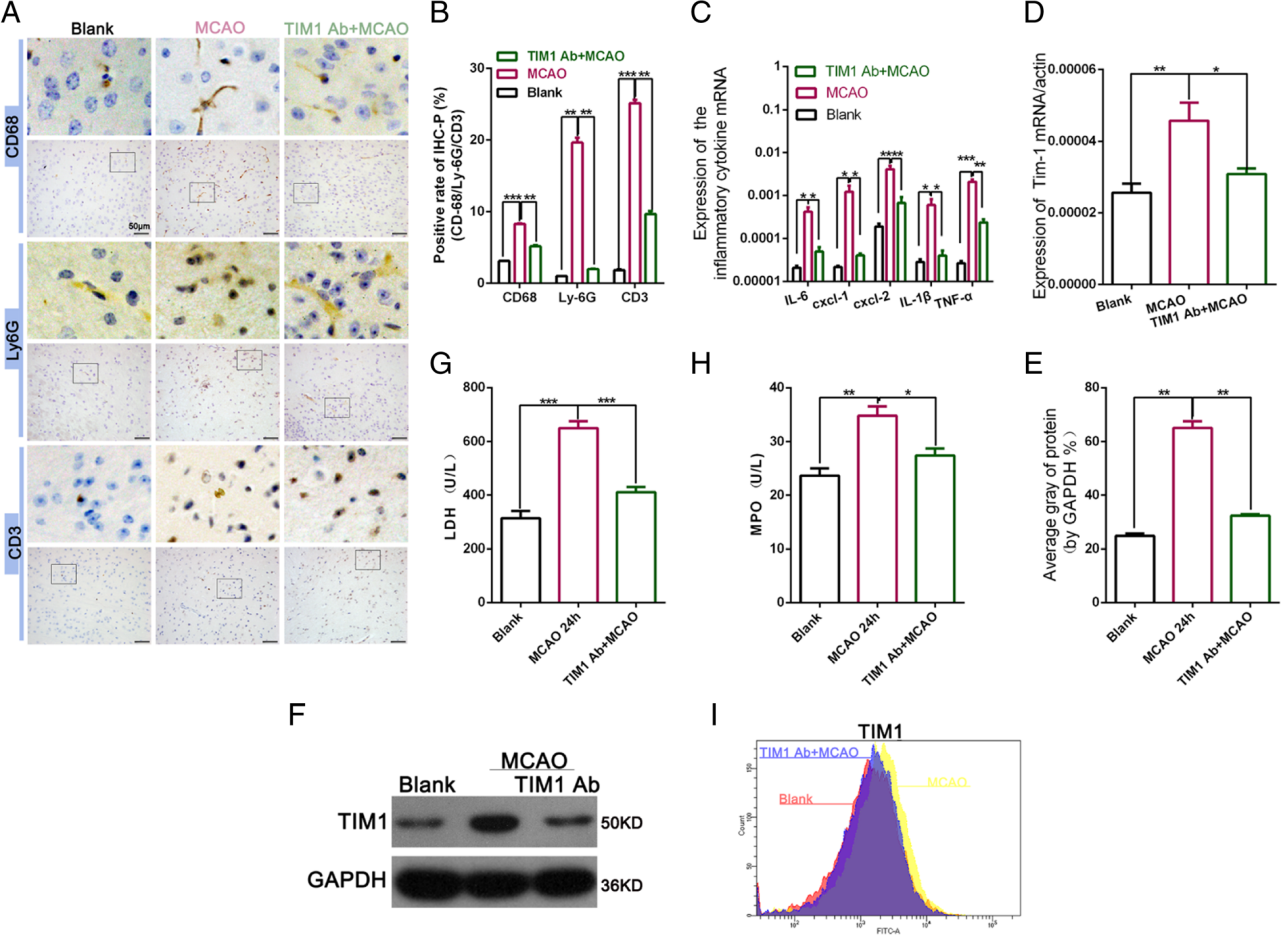
TIM-1 disruption effectively reduced neutrophil and macrophage function in the brain tissue. **a** and **b** Immunohistochemical staining was used to analyze the number of CD3 and CD68 macrophages, as well as Ly6G neutrophils. **c** qRT-PCR was used to examine the expression of interleukin (IL-6), chemokine (C-X-C motif) ligand 1 (CXCL1), CXCL-2, IL-1β, and tumor necrosis factor alpha (TNF-α). **p < 0.01; ***p < 0.001. **d** qRT-PCR was used to detect the level of TIM-1 expression following treatment with or without an anti-TIM-1 Ab in the MCAC model or Black group. *p < 0.05; **p < 0.01. **e** and **f** Western blot analysis of the level of TIM-1 expression following treatment with or without an anti-TIM-1 Ab in the MCAC model or Black group. **p < 0.01. **g** and **h** An ELISA was used to examine the level of MPO and LDH expression. **i** Flow cytometry detection of TIM-1 expression

The inflammatory response is the main factor inducing nerve cell injury after MCAO, which plays an important role in MCAO injury [21]. To further explore the role of the TIM-1 blockade in the MCAO model, we used an immunohistochemical analysis to analyze the migration of neutrophils and macrophages in MCAO. The results found that blocking TIM-1 substantially reduced CD3, the frequency of CD68 macrophages, and the number of ly6G neutrophils (Fig. 3a-b). We also examined the level of neutrophil/monocyte-derived pro-inflammatory chemokines after inhibiting TIM-1. We detected the graft expression of cytokines (IL- 6, IL-1β, and TNF-α) and chemokines (CXCL-1 and CXCL-2), which were analyzed using qRT-PCR. Compared with the MCAO group, an inhibition of TIM-1 resulted in a significantly decreased induction of IL-6, IL-1β, and TNF-α (Fig. 3c). CXCL-1 and CXCL-2 predominantly drives neutrophil trafficking [22]. The reduced expression of CXCL-1 and CXCL-2 also suggest decreased neutrophil infiltration (Fig. 3c). QRT-PCR and a Western blot analysis showed that blocking TIM-1 in an MCAO model could down-regulate the increased TIM-1 expression caused by MCAO (Fig. 3d-f). An MPO assay, reflecting neutrophil activity (U/L), was suppressed in the anti-TIM-1 mAb + MCAO treatment group compared with MCAO. Furthermore, LDH and MPO were significantly increased in MCAO compared with the Blank group whereas they significantly decreased following TIM-1 inhibition compared with MCAO (Fig. 3g-h). Flow cytometry analysis of TIM-1 expression on the surface of T cells revealed that treatment with an anti-TIM-1 mAb in the MCAO model could reduce TIM-1 expression compared with MCAO (Fig. 3i).

### Neuronal cell damage mediated by T cells was significantly reduced following treatment with an anti-TIM-1 mAb

**Fig. 4:**
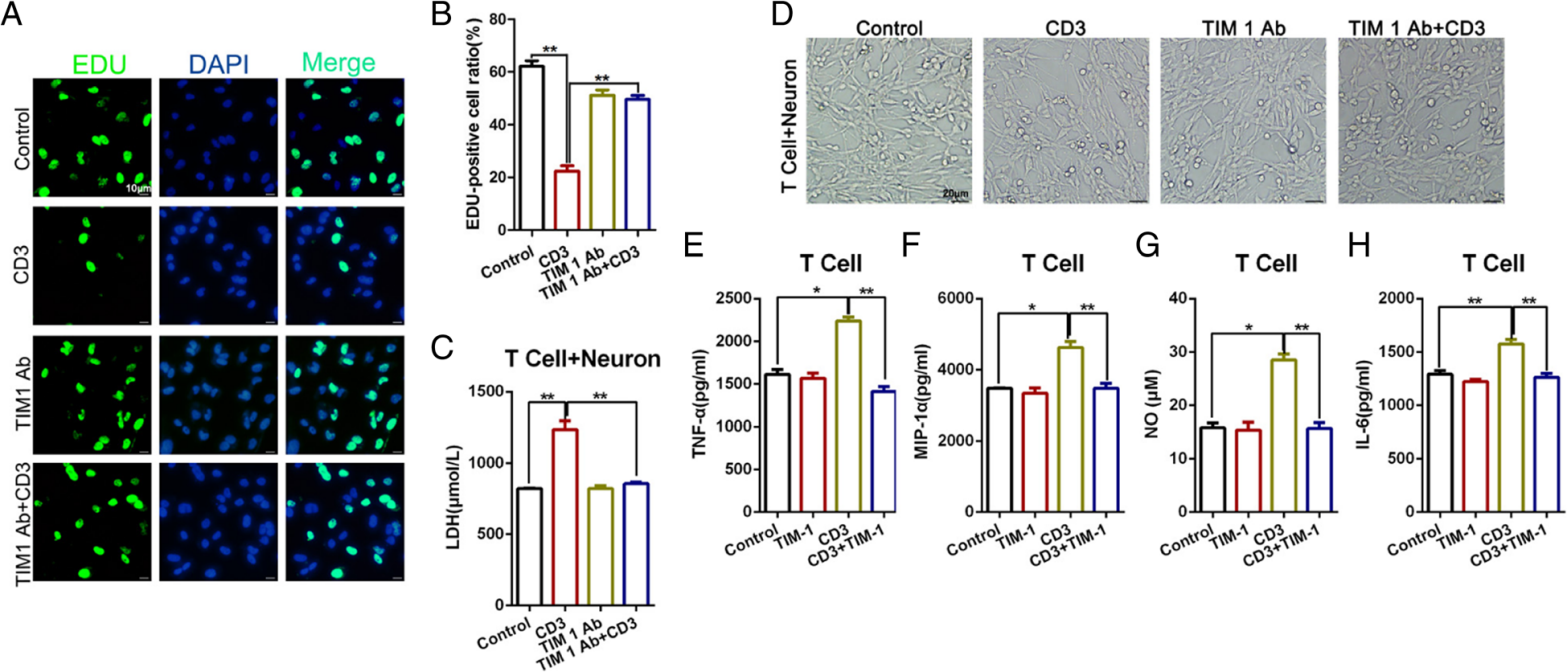
The level of T cell-mediated damage on neurons was significantly reduced following in vitro treatment of co-cultured T cells and neurons with an anti-TIM-1 antibody. **a**-**b** Representative images and quantification of an Edu incorporation assay for cellular proliferation. **p < 0.01. **c** LDH expression from T cell + neuron cells in each of the different treatment groups. **p < 0.01. **d** Light microscopy for detecting neuronal cell morphology. **e**-**h** An ELISA was used to examine the changes in cytokine secretion, including TNF-α, MIP-1α, NO, and IL-6 in each group. *p < 0.05; **p < 0.01

To identify the effect of TIM-1 on neurons, we used CD3 magnetic beads to separate the T cells from the peripheral blood, then co-cultured the isolated cells with the neuronal cells. We then used CD3, anti-TIM-1 mAbs, and CD3 combined with anti-TIM-1 mAbs to stimulate the cells. An EdU assay indicated that the level of cellular proliferation was decreased in response to CD3 stimulation, whereas proliferation increased in the group treated with the anti-TIM-1 mAbs combined with CD3, compared with the CD3-treated group alone (Fig. 4a-b). An ELISA was used to determine the expression of LDH to evaluate the neuronal cell activity. The resultes indicated that the most severe damage to the neuronal cells occurred in the CD3 group, whereas the group treated with TIM-1 combined with CD3 displayed reduced damage compared with the CD3 group; however, there was no significant difference between the TIM-1 mAb-treated group and the TIM-1 Ab + CD3 group (Fig. 4c). Furthermore, we used light microscopy to detect the morphology of the total neuronal cells in each treatment group (Fig. 4d). Mechanistically, we examined the changes in the secreted cytokines, including TNF-α, MIP-1α, NO, and IL-6 in each group with an ELISA. We found that stimulation with CD3 could increase the expression of TNF-α, MIP-1α, NO, and IL-6, whereas anti-TIM-1 mAbs could decrease such expression (Fig. 4e-h). These findings indicate that a TIM-1 blockade could significantly reduce the T cell-mediated damage to neurons when T cells and neurons are co-cultured in vitro.

### Following TIM-1 inhibition, the T cell-mediated neuron damage was significantly reduced when T cells and neurons were co-cultured in vitro

**Fig. 5:**
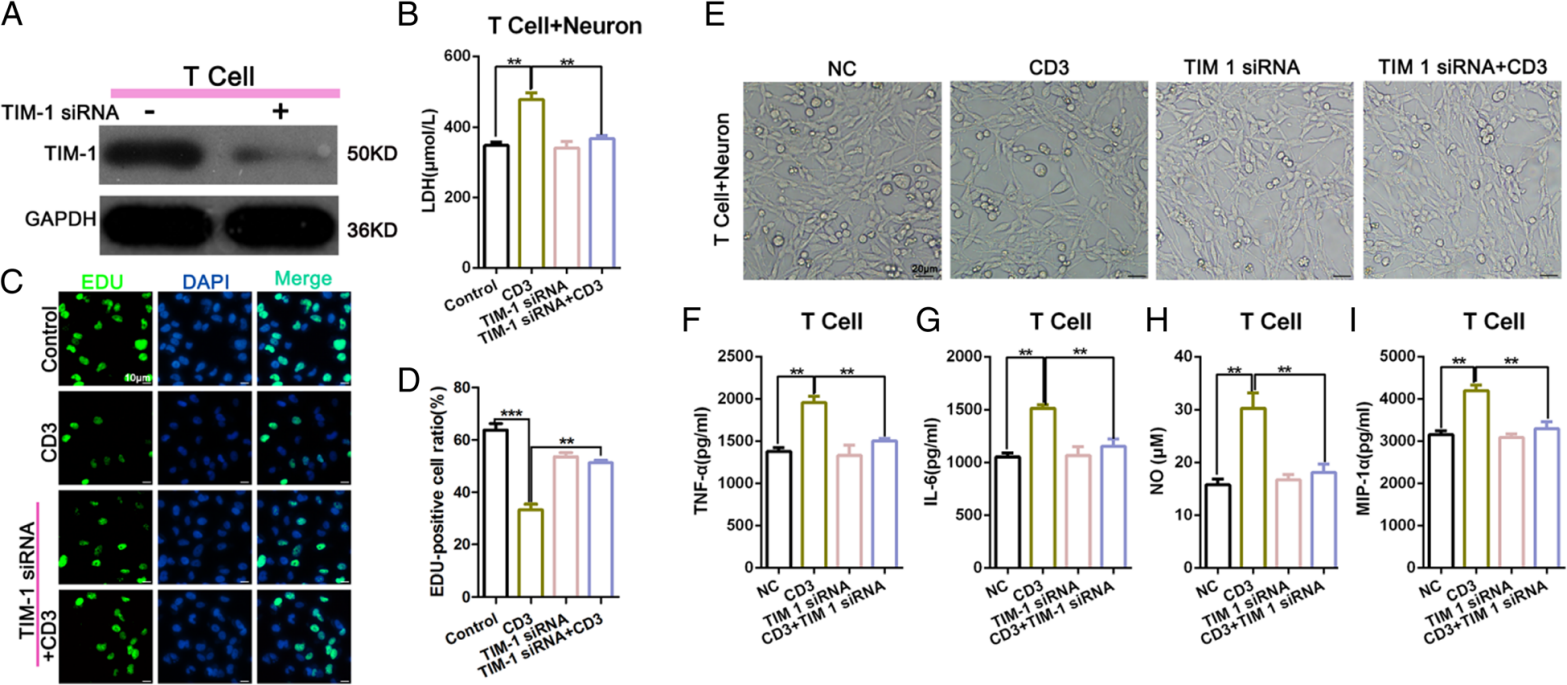
The level of T cell-mediated damage on neurons was significantly reduced following an in vitro TIM-1 knockdown in co-cultured T cells and neurons. **a** Western Blot detection of the TIM-1 transfection efficiency. **b** The level of LDH expression assessed by an ELISA.**p < 0.01. **c**-**d** An Edu incorporation assay was used to examine the level of cellular proliferation. Representative images and quantification was represented by histogram. **p < 0.01; ***p < 0.001. **e** Light microscopy for the detection of neuronal cell morphology. **f**-**i** The detection of cytokines secreted, including TNF-α, MIP-1α, NO, and IL-6 in each group by ELISA. **p < 0.01

Next, to determine the mechanism of T cell-mediated damage on neurons, we knocked down TIM-1 and used a Western blot to detect the interference efficiency (Fig. 5a). The ELISA results indicated that the expression of LDH in the CD3 group was higher than that in the Control group. There was no significant difference between TIM-1 expression in the siRNA group and TIM-1 in the siRNA + CD3 group (Fig. 5b). The EdU assay showed that the level of TIM-1 siRNA combined with CD3 could enhance the decrease in cellular proliferation observed in the CD3 group (Fig. 5c-d). Light microscopy was used to examine the morphology of the total neuronal cells in each treatment group (Fig. 5e). We performed an ELISA to examine the changes in cytokine secretion, including the level of TNF-α, IL-6, NO, and MIP-1α in each group with an ELISA. We found that CD3 stimulation could increase the expression of TNF-α, MIP-1α, NO, and IL-6, whereas blocking TIM-1 decreased such production (Fig. 5f-i). These findings indicate that the knockdown of TIM-1 could significantly reduce the T cell-mediated damage to neurons when T cells and neurons were co-cultured in vitro.

## Discussion

TIM-1 is a type I cell-surface glycoprotein with an immunoglobulin V-like domain, a mucin domain, a single transmembrane region, and a cytoplasmic tail containing a tyrosine phosphorylation motif [10, 23]. TIM1 is an endogenous ligand for leukocyte mono-immunoglobulin (Ig)–like receptor 5 (LMIR5)/CD300b, and soluble TIM1 through both direct and indirect mechanisms to induce the neutrophil accumulation via LMIR5 under in vivo conditions, which was involved in the tubular damage in the acute phase after IRI [24]. Furthermore, TIM1 was associated with human cancer, it has reported that activation of TIM1 by exposing the cells to TIM4 significantly increased colon cancer cell apoptosis through up-regulating FasL level [25]. Recently, an important role for TIM-1 costimulatory signaling in native T cell differentiation, proliferation, and macrophage activation has been revealed. Increasing evidence indicates that TIM-1 signaling plays an important role in I/R injury (e.g., renal and liver), by activating T cells and aggravating the tissue damage [18, 24]. Interestingly, since T cells constitute one of the primary arms of the adaptive immune response, the inhibition of T cell activities has been developed for stroke treatment [26]. To the best of our knowledge, the expression and function of TIM-1 in the process of cerebral ischemia-reperfusion injury have not been previously reported.

The role of myeloid leukocytes have been explored during IRI progression and are considered to be primarily responsible for IRI [12]. More recently, the essential role of TIM-1 costimulatory signals associated with T cell differentiation, proliferation, and macrophage activation has been revealed. In a mouse IRI model, the TIM-1 signaling pathway has been shown to be related to IRI hepatocyte injury through increasing the number of T cells, neutrophils, and macrophages; in the direct pathway, TIM-1 on activated Th2 cells cross-links TIM-4 to directly activate macrophages; in the indirect pathway, TIM-1 on activated Th1 cells triggers IFN-γ production leaded to macrophage activation [27]. TIM1 is co-localized with CD3 on the surface of T-cell, it may be functional as part of the T-cell receptor (TCR) signaling complex during T-cell activation, possibly through IL-2-induced T-cell kinase (ITK) and phosphoinositide 3-kinase (PI3K) phosphorylation [10, 28, 29]. It has been previously reported that targeting TIM-1 expression on CD4 positive cells could suppress macrophage activation, resulting in decreased IRI [30]. In the present study, we found that the level of TIM-1 was up-regulated at 24 h and 48 h after MCAO by qRT-PCR and Western blot, respectively. Furthermore, TIM-1 expression was increased following CD3 monoclonal stimulation. As indicated by the results of the Western blot and flow cytometry analysis, CD3 monoclonal stimulation could directly regulate TIM-1expression. Furthermore, we further demonstrated that TIM-1 inhibition using anti-TIM-1 mAbs or TIM-1 siRNA could significantly reduce the T cell-mediated damage to neurons when T cells were co-cultured with neuronal cells in vitro. These results suggest that the level of TIM-1 was related to T cell activation and it might be a promising MCAO biomarker.

Apoptosis and the inflammatory response play an important role in cerebral I/R injury, and are the main factors inducing nerve cell injury following I/R [21, 31]. The increased infarct size was correlated with more severe neurobehavioral deficits in the MCAO injury mice according to the Bederson scores. However, treatment with anti-TIM-1 mAbs reduced the infarct size compared with the MCAO group, suggesting that TIM-1 may play a protective role in MCAO. Treatment with the TIM-1 mAb MCAO demonstrated reduced cellular apoptosis with a TUNEL assay; moreover, it could down-regulate Bax and up-regulate the expression of Bcl-2 and Bcl-xl compared with the MCAO group, suggesting that a TIM-1 blockade might downregulate apoptotic pathways; however, contrary to these studies, we observed a reduction in the number of apoptotic cells within MCAO after blocking TIM-1, indicating that TIM-1 may be involved with the number of apoptotic cells. It has been reported that TIM-1 may directly regulate Tbet and FoxP3, the key Th1 and Treg transcription factors in CD4 Th cell differentiation, respectively to facilitate downstream signature biomarkers of liver damage (i.e. IFN-γ; TNF-α) or cytoprotection (i.e. IL-6;IL-10) [14, 15, 32]. Cytokines and chemokines, including IL- 6, IL-1β, and TNF-α, CXCL-1, and CXCL-2 have been shown to directly recruit and activate T cells [33]. We found a sustained upregulation in the level of cytokines and chemokines after MCAO, indicating that these cytokines are potential mediators involved in the pathogenesis that occurs following MCAO injury. However, the TIM-1 blockade could significantly decrease the expression of IL-6, IL-1β, and TNF-α, CXCL-1, and CXCL-2, indicating that the inhibition of TIM-1 primarily suppressed macrophage activation. These findings suggest that the local function of these cytokines could be crucial for the pathogenesis observed following I/R injury, which is consistent with the reported results [34]. An immunohistochemical analysis showed that the inhibition of TIM-1 substantially reduced CD3, the frequency of CD68 macrophages, and the number of ly6G neutrophils. These data indicate that interfering with TIM-1 co-stimulation is a novel therapeutic approach for managing T cell activation and inflammation in MCAO.

## Conclusion

In conclusion, MCAO injury triggers TIM-1 signaling to activate T cells, which in turn, has a downstream effect on later inflammation and organ dysfunction. A TIM-1 blockade protects against MCAO injury by inhibiting T cell activation, leading to the decreased attenuation of apoptosis. This study provides evidence for a novel mechanism by which TIM-1 signaling affects innate immunity-driven inflammatory responses during the course of MCAO injury. Therefore, disrupting TIM-1 may serve as a novel target for therapy following MCAO.

## Data Availability

All data generated or analyzed during this study are included in this published article.

## Abbreviations

I/R): Ischemic reperfusion
IL-6): Interleukin
LDH): Lactate dehydrogenase
MCAO): Middle cerebral artery occlusion
MIP-1α): Macrophage inflammatory protein-1α
Th2): T-helper 2
TIM): T cell immunoglobulin and mucin domain
TNF-α): Tumor necrosis factor alpha
TUNEL): TdT-mediated biotin-16-dUTP nick-end labeling
