# Correction to: Inhibition of T cell immunoglobulin and mucin-1 (TIM-1) protects against cerebral ischemia-reperfusion injury

**DOI:** 10.1186/s12964-019-0427-2

**Published:** 2019-09-02

**Authors:** Yueying Zheng, Liqing Wang, Manli Chen, Lu Liu, Aijie Pei, Rong Zhang, Shuyuan Gan, Shengmei Zhu

**Affiliations:** Department of Anesthesiology, The 1st Affiliated Hospital, School of Medicine, Zhejiang University, 79# Qingchun Road, Hangzhou, Zhejiang Province 310003 People’s Republic of China

**Correction to: Cell Commun Signal**

**https://doi.org/10.1186/s12964-019-0417-4**

Following publication of the original article [1], the authors reported that the given name of Liqing Wang was incorrectly published as Liqiang Wang. The original article has been updated.
